# Supplementary material for: Functional investigation suggests CNTNAP5 involvement in glaucomatous neurodegeneration obtained from a GWAS in primary angle closure glaucoma
Source: PLoS Genet. 2024 Dec 5;20(12):e1011502. doi: 10.1371/journal.pgen.1011502 (PMC11651621; doi:10.1371/journal.pgen.1011502)
Supplement: S1 Table — (DOCX) [file pgen.1011502.s001.docx]

| SNP | P-value | OR | 95% CI |
| --- | --- | --- | --- |
| rs17011381 | 8.86E-06 | 2.5 | 1.90-3.28 |
| rs2901264 | 9.37E-06 | 0.65 | 0.48-0.88 |
| rs2115890 | 7.12E-06 | 2.8 | 2.00-3.91 |
| rs1430263 | 3.37E-06 | 0.72 | 0.60-0.86 |
| rs17011394 | 9.87E-06 | 0.58 | 0.42-0.80 |
| rs780010 | 2.13E-06 | 2.94 | 2.01-4.30 |
| rs17724018 | 2.52E-06 | 1.45 | 0.92-1.73 |
| rs779979 | 4.27E-06 | 0.68 | 0.55-0.84 |
| rs2553625 | 5.09E-06 | 1.9 | 1.40-2.58 |
| rs17011420 | 5.36E-06 | 2.8 | 2.10-3.72 |
| rs2553628 | 4.86E-06 | 1.58 | 1.08-2.45 |
| rs17011429 | 5.86E-06 | 1.85 | 1.28-2.72 |

**S1_Table:** The table presents the association between 13 SNPs of *CNTNAP5* and PACG, showing P-values, odds ratios (OR), and 95% confidence intervals (CI).
