# Supplementary material for: Functional investigation suggests CNTNAP5 involvement in glaucomatous neurodegeneration obtained from a GWAS in primary angle closure glaucoma
Source: PLoS Genet. 2024 Dec 5;20(12):e1011502. doi: 10.1371/journal.pgen.1011502 (PMC11651621; doi:10.1371/journal.pgen.1011502)
Supplement: S2 Table — (DOCX) [file pgen.1011502.s002.docx]

| Morpholino | Sequence |
| --- | --- |
| *cntnap5a* | TTAAGATTTGGTCTTGTTGGCATCT |
| *cntnap5a*_5_base_mismatch | TTAAcATTTcGTgTTcTTGcCATCT |
| *cntnap5b* | GGCCGAGCGAAGATATTCCATGTTC |
| *cntnap5b*_5_base_mismatch | GGCCcAcCGAAcATATTCgATcTTC |

**S2_Table**: Morpholino sequence of cntnap5a, cntnap5b and their respective mismatch 5 base mismatch control.
