# Supplementary material for: Functional investigation suggests CNTNAP5 involvement in glaucomatous neurodegeneration obtained from a GWAS in primary angle closure glaucoma
Source: PLoS Genet. 2024 Dec 5;20(12):e1011502. doi: 10.1371/journal.pgen.1011502 (PMC11651621; doi:10.1371/journal.pgen.1011502)
Supplement: S3 Table — (DOCX) [file pgen.1011502.s003.docx]

| Chromosome | SNP | RegulomeDB Rank |
| --- | --- | --- |
| 2 | rs17011420 | 3a |
| 2 | rs17724018 | 4 |
| 2 | rs2115890 | 4 |
| 2 | rs17011429 | 5 |
| 2 | rs2901264 | 5 |
| 2 | rs1430263 | 5 |
| 2 | rs779979 | 5 |
| 2 | rs17011399 | 6 |
| 2 | rs780010 | 6 |
| 2 | rs733112 | 6 |
| 2 | rs2553625 | 7 |

**S3_Table:** RegulomeDB score of 13 SNPs of *CNTNAP5*
