# Supplementary material for: Functional investigation suggests CNTNAP5 involvement in glaucomatous neurodegeneration obtained from a GWAS in primary angle closure glaucoma
Source: PLoS Genet. 2024 Dec 5;20(12):e1011502. doi: 10.1371/journal.pgen.1011502 (PMC11651621; doi:10.1371/journal.pgen.1011502)
Supplement: S4 Table — Each row includes the Variant ID, chromosome location (Chr), nominal p-value indicating the statistical significance of the association, r_squared (coefficient of determination) representing the proportion of variance explained by the variant, beta (slope) indicating the direction and magnitude of the effect on CNTNAP5 expression, and slope_se representing the standard error of the beta estimate. Variants with lower nominal p-values suggest stronger evidence of association with CNTNAP5 expression. (DOCX) [file pgen.1011502.s004.docx]

| **Variant_ID** | **Chr** | **nominal P value** | **r_squared** | **beta (slope)** | **slope_se** |
| --- | --- | --- | --- | --- | --- |
| rs17011381 | chr2 | 0.00930 | 0.01675 | 0.37025 | 0.14166 |
| rs2901264 | chr2 | 0.25371 | 0.00325 | -0.11959 | 0.10463 |
| rs2115890 | chr2 | 0.10104 | 0.00669 | -0.17931 | 0.10910 |
| rs1430263 | chr2 | 0.87580 | 0.00006 | -0.01890 | 0.12085 |
| rs17011394 | chr2 | 0.87107 | 0.00007 | -0.01969 | 0.12126 |
| rs780010 | chr2 | 0.16974 | 0.00045 | 0.04624 | 0.10835 |
| rs17724018 | chr2 | 0.43341 | 0.00153 | -0.17669 | 0.22532 |
| rs779979 | chr2 | 0.38316 | 0.00190 | 0.08716 | 0.09983 |
| rs2553625 | chr2 | 0.04423 | 0.02142 | 0.33398 | 0.11274 |
| rs17011420 | chr2 | 0.00365 | 0.00730 | 0.17926 | 0.10437 |
| rs2553628 | chr2 | 0.00529 | 0.00534 | 0.15117 | 0.10300 |
| rs17011429 | chr2 | 0.00522 | 0.01930 | 0.31897 | 0.11356 |

**S4_Table. Summary of eQTL Analysis for 13 SNPs at the *CNTNAP5* Locus:** Table represents the eQTL analysis results for 13 SNPs associated with *CNTNAP5* expression. Each row includes the Variant ID, chromosome location (Chr), nominal p-value indicating the statistical significance of the association, r_squared (coefficient of determination) representing the proportion of variance explained by the variant, beta (slope) indicating the direction and magnitude of the effect on *CNTNAP5* expression, and slope_se representing the standard error of the beta estimate. Variants with lower nominal p-values suggest stronger evidence of association with *CNTNAP5* expression.
