# Supplementary material for: Functional investigation suggests CNTNAP5 involvement in glaucomatous neurodegeneration obtained from a GWAS in primary angle closure glaucoma
Source: PLoS Genet. 2024 Dec 5;20(12):e1011502. doi: 10.1371/journal.pgen.1011502 (PMC11651621; doi:10.1371/journal.pgen.1011502)
Supplement: S5 Table — These SNPs that were distributed in two LD blocks (the SNPs in pink belong to one LD block and the ones in green belonged to the other LD block) and showed similar observed and expected read counts. (DOCX) [file pgen.1011502.s005.docx]

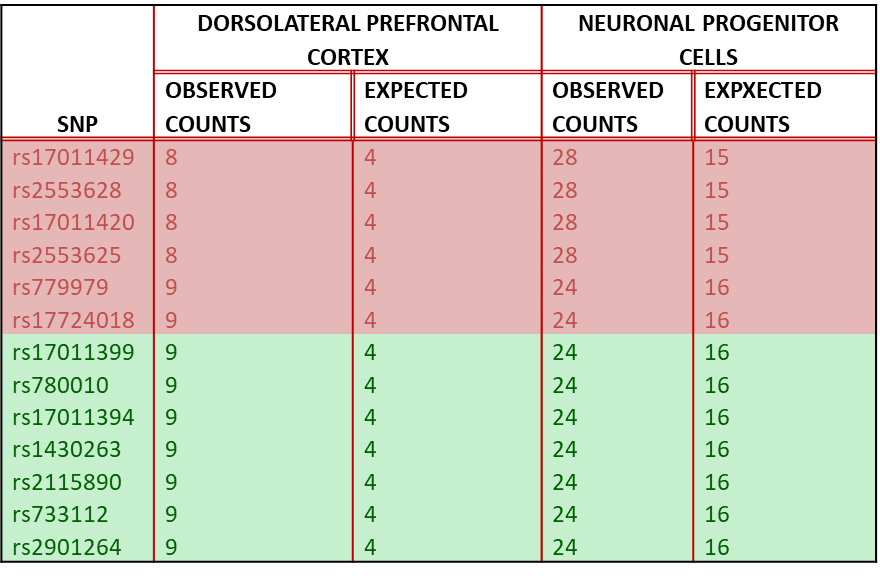


**S5_Table**: Observed and expected counts of the intronic SNPs of *CNTNAP5* found from HUGIn. These SNPs that were distributed in two LD blocks (the SNPs in pink belong to one LD block and the ones in green belonged to the other LD block) and showed similar observed and expected read counts.
