# Supplementary material for: Functional investigation suggests CNTNAP5 involvement in glaucomatous neurodegeneration obtained from a GWAS in primary angle closure glaucoma
Source: PLoS Genet. 2024 Dec 5;20(12):e1011502. doi: 10.1371/journal.pgen.1011502 (PMC11651621; doi:10.1371/journal.pgen.1011502)
Supplement: S6 Table — The gene also showed low but measurable confidence scores for epilepsy and retinal disease. Other neurodegenerative diseases like Parkinson’s and Alzheimer’s showed very low confidence scores. (DOCX) [file pgen.1011502.s006.docx]

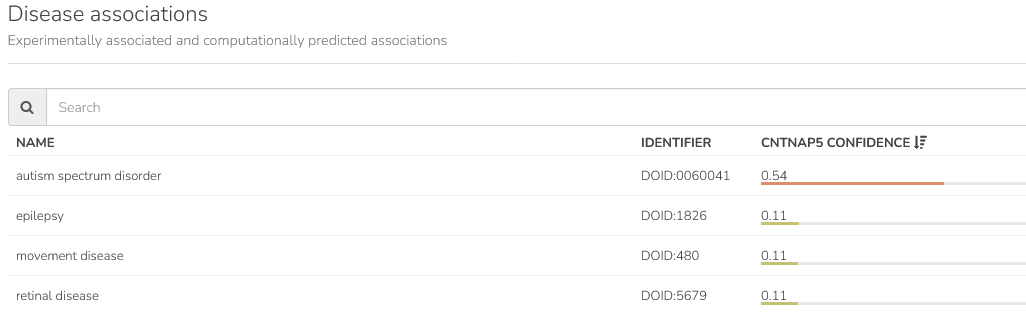


**S6_Table**: HumanBase portal showed *CNTNAP5* as the highest confidence score for autism spectrum disorder. The gene also showed low but measurable confidence scores for epilepsy and retinal disease. Other neurodegenerative diseases like Parkinson's and Alzheimer's showed very low confidence scores. This table is a screenshot from the HumanBase portal <https://hb.flatironinstitute.org/>.
