# Supplementary figures and images for: Functional investigation suggests CNTNAP5 involvement in glaucomatous neurodegeneration obtained from a GWAS in primary angle closure glaucoma
Source: PLoS Genet. 2024 Dec 5;20(12):e1011502. doi: 10.1371/journal.pgen.1011502 (PMC11651621; doi:10.1371/journal.pgen.1011502)

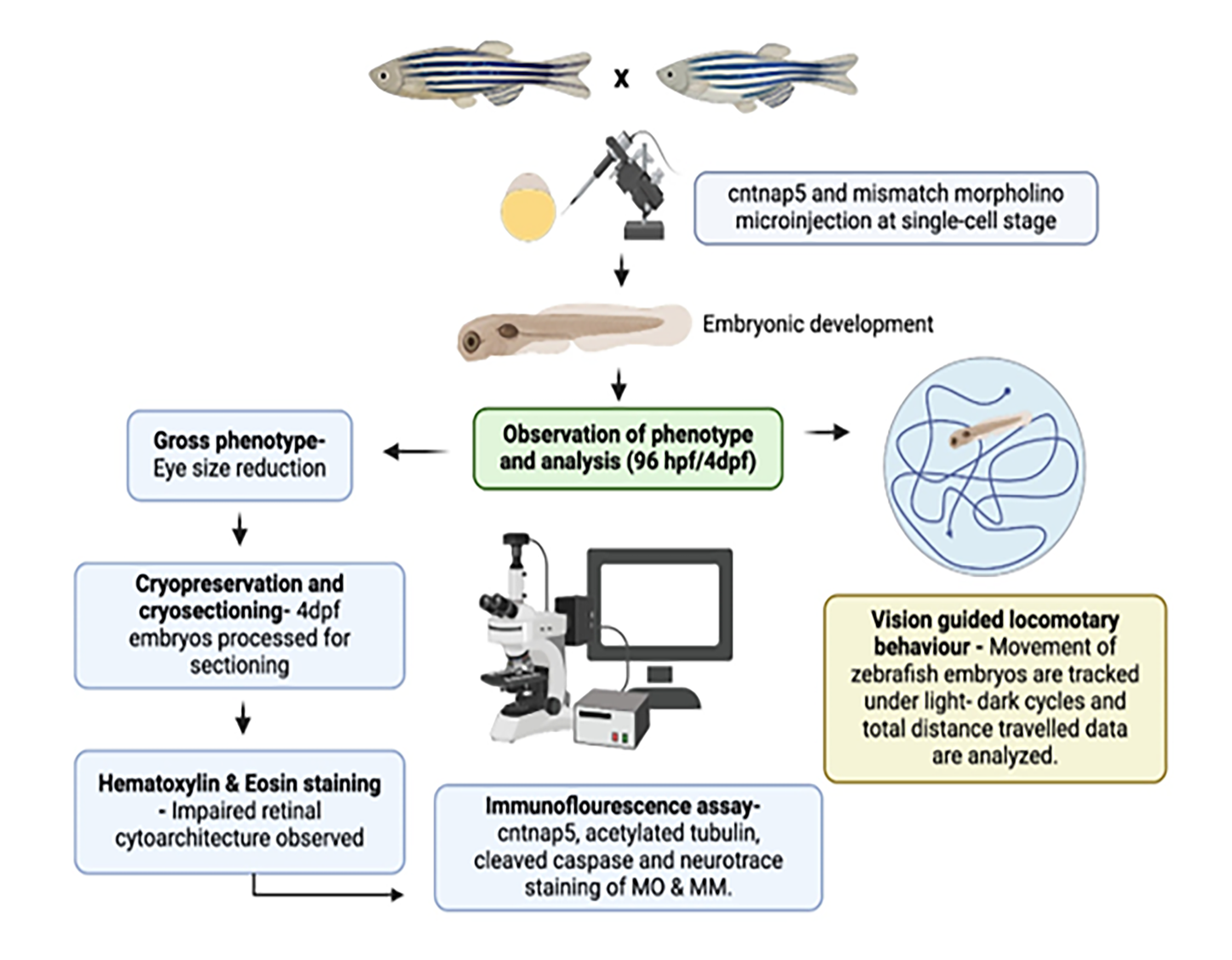

Supplement: S1 Fig — Part of this figure is created with BioRender.com, a licenced version to users of the National Institute of Biomedical Genomics, India. (TIF) [file pgen.1011502.s007.tif]

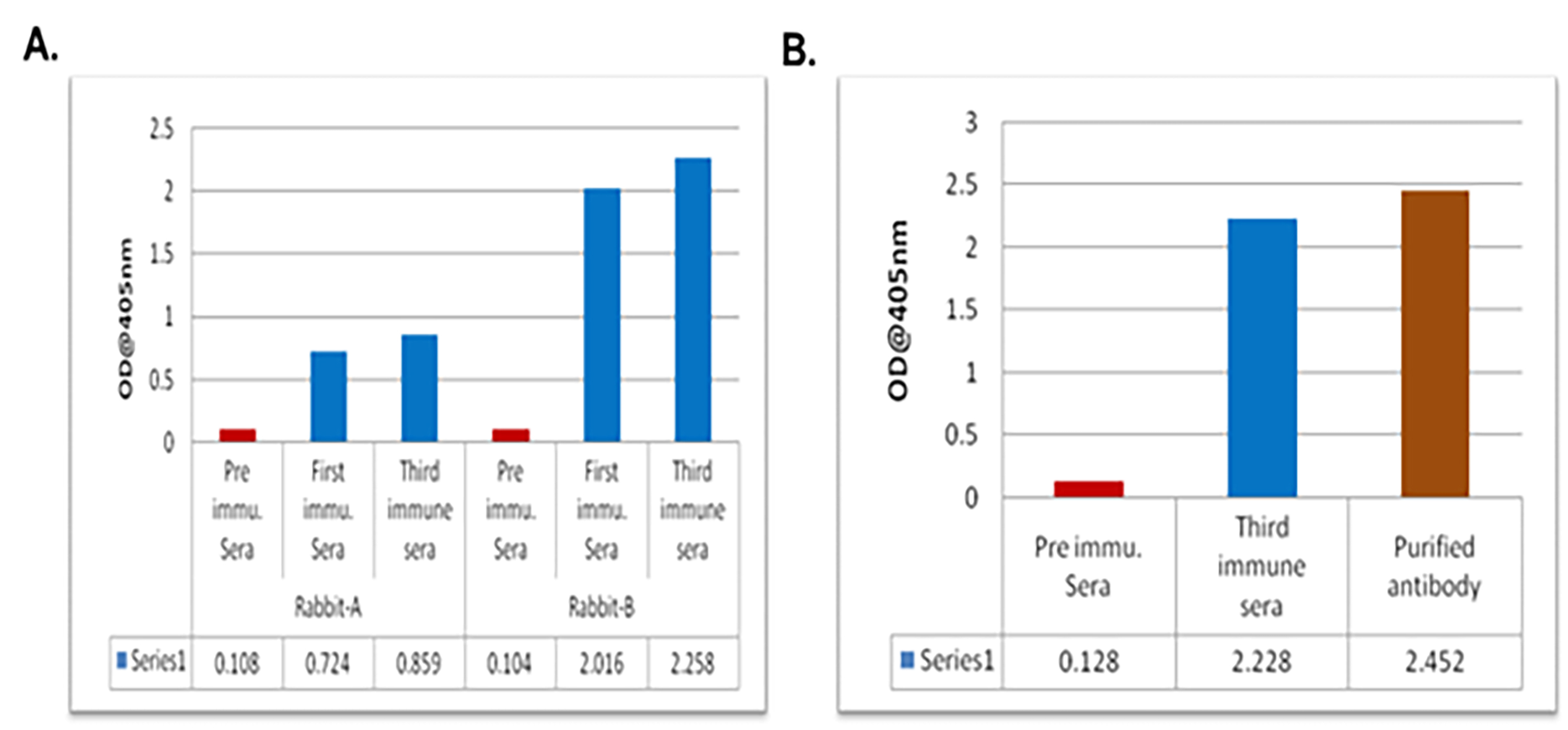

Supplement: S2 Fig — A. First and Third immune sera of Rabbit A and B tested against the antigen coated at 200ng/well and at 1:5000 dilutions of primary Ab. Pre-immune sera were used as control in place of primary antibody. Plates read after 15 min of enzyme substrate reaction and the absorbance were measured at 405nm. B. Immune sera (at 1:5000) and Purified antibody (at 200ng/well) tested against the antigen coated at 200ng/well obtained a value of 2.228 and 2.452 respectively. Pre-immune sera were used as control in place of primary antibody. Plates read after 15 min of enzyme substrate reaction and the absorbance were measured at 405nm. (JPG) [file pgen.1011502.s008.jpg]

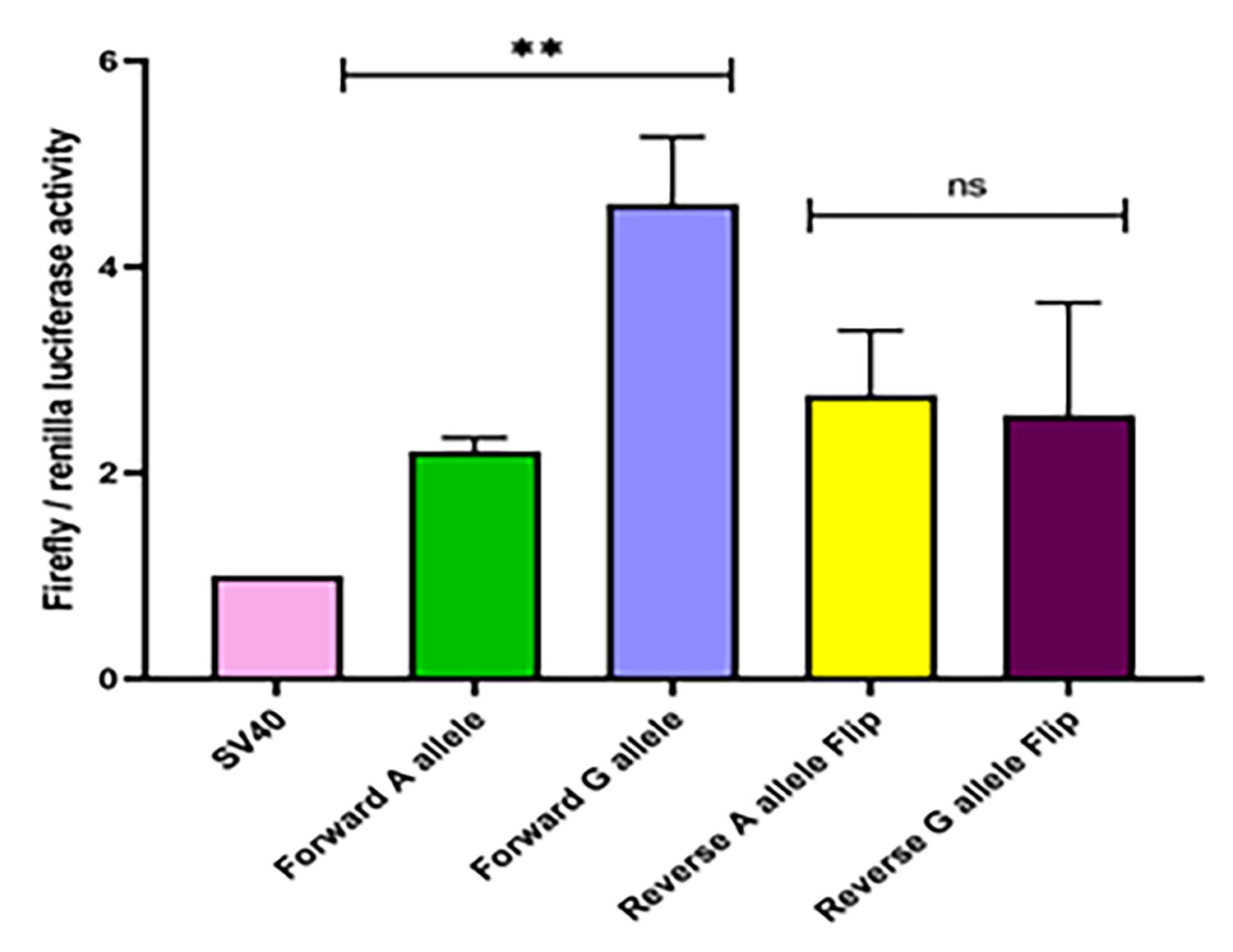

Supplement: S3 Fig — The bar plots the mean value of all the 3 experiments, while error bars are depicting SD. Two tailed student t-test for independent means was used for calculating statistical significance; *p < 0.05, **p < 0.01; ***p < 0.001, ns- = not significant. The luciferase assay shows G allele of rs2553628(CNTNAP5) has a statistically significant higher firefly / renilla luciferase activity than the A allele of rs2553628 (CNTNAP5) for both forward orientation and reverse orientation. P-value: 0.0034 (Forward set) P-value: 0.8041 (Reverse set). (TIF) [file pgen.1011502.s009.tif]

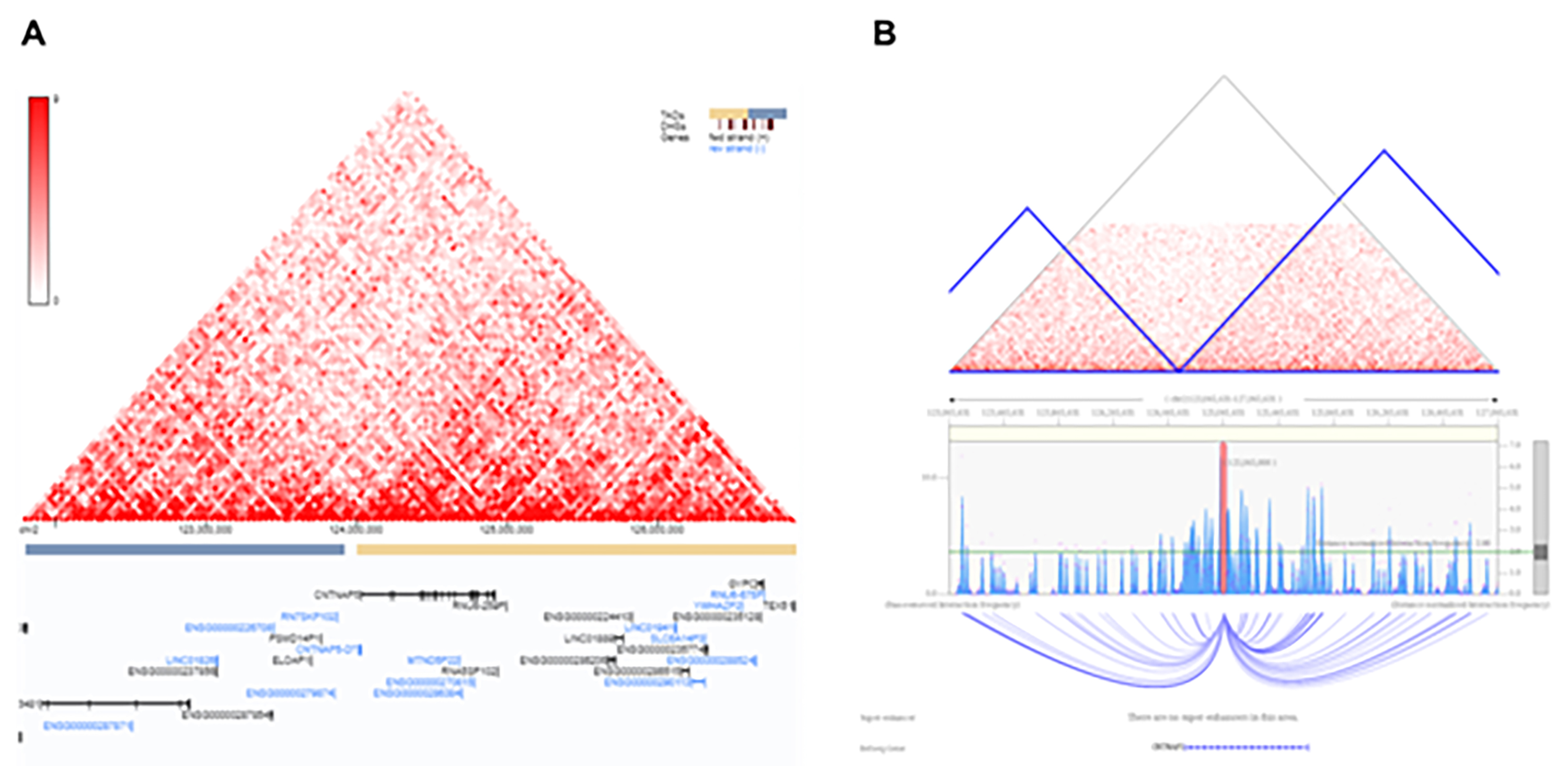

Supplement: S4 Fig — A. The Hi-C heatmaps clearly show strong TAD correlation around genomic region chr2:125,083,095–125,084,384 of CNTNAP5. B. Visualization of significant long-range chromatin interactions centered on the genomic coordinates chr2:125,083,095–125,084,384 located in the CNTNAP5 intron. The figure is organized from top to bottom as follows: 1 Normalized Hi-C contact map with TAD annotations (blue triangles). 2 Identified chromatin interactions, where arcs represent long-range interactions between genomic regions. 3 RefSeq genes in the region. 4 Interaction frequency graphs: o Blue bar graph: Bias-removed interaction frequencies. o Magenta dots: Distance-normalized interaction frequencies. o Green threshold line: Interactions with blue bars above this line are considered significant (2-fold greater than the background). The x-axis represents the genomic position, with the center focusing on the CNTNAP5 intron region. Proximal interactions are shown closer to the center, while distal interactions extend further from the center. This visualization helps identify potential regulatory interactions between the CNTNAP5 intron and other genomic regions, both nearby (proximal) and far away (distal). (JPG) [file pgen.1011502.s010.jpg]

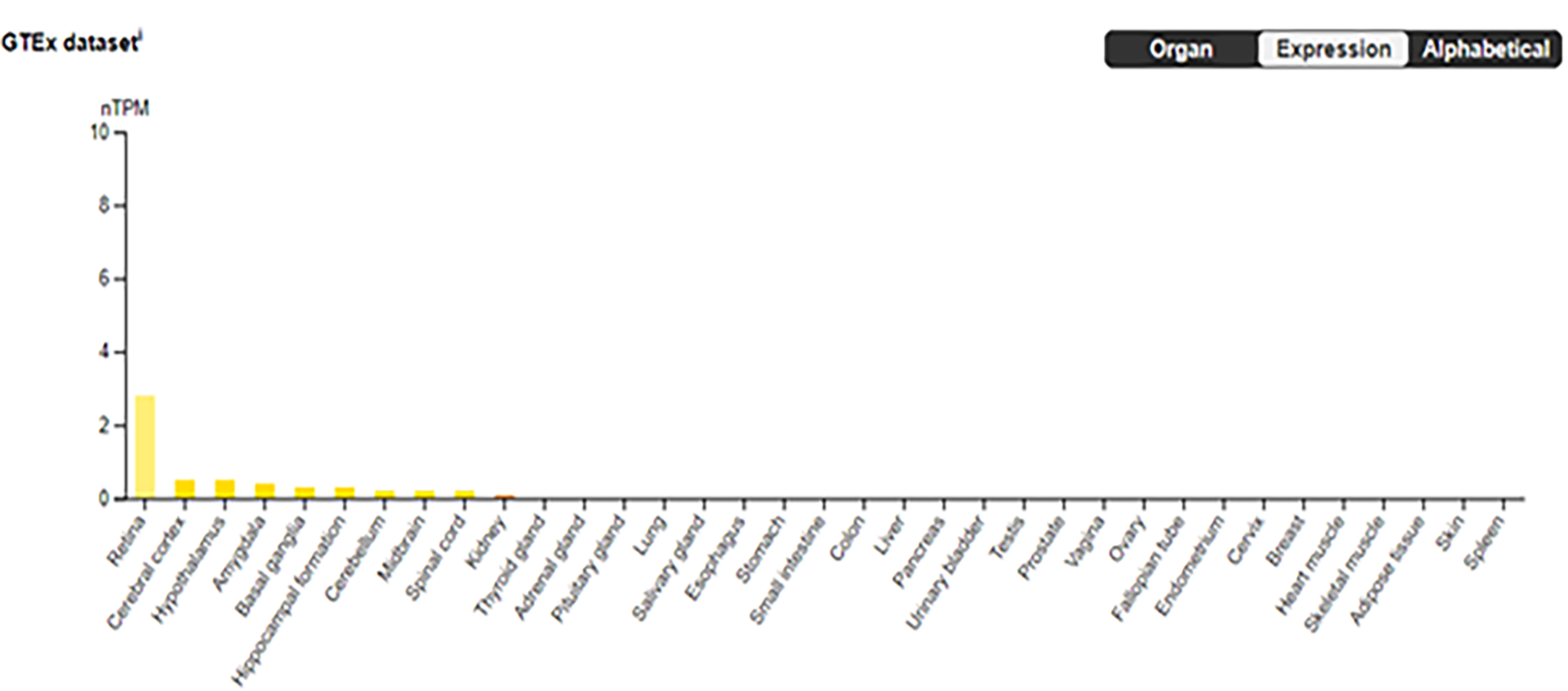

Supplement: S5 Fig — (JPG) [file pgen.1011502.s011.jpg]

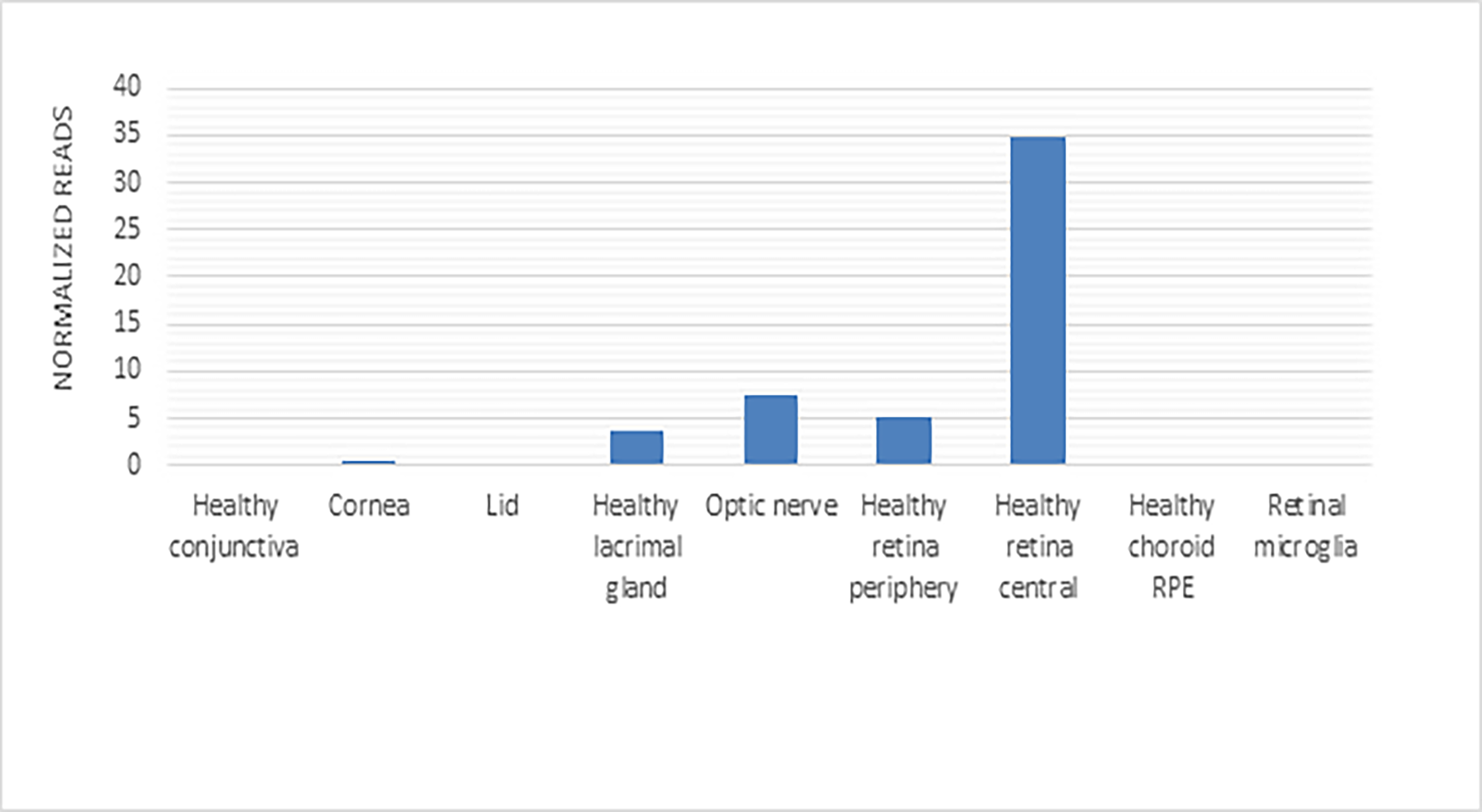

Supplement: S6 Fig — (TIF) [file pgen.1011502.s012.tif]

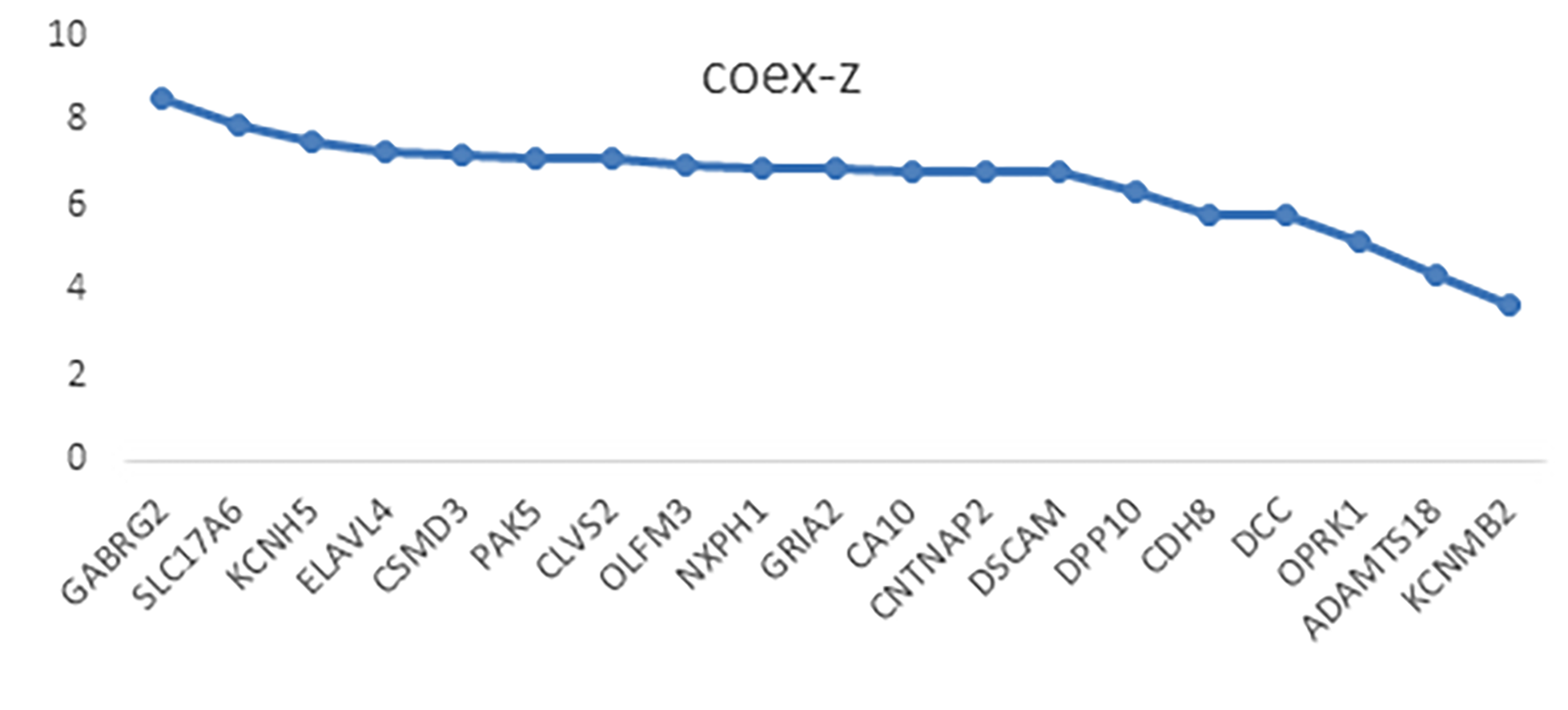

Supplement: S7 Fig — Co-expression values as coex-z (a coex z values) is greater than 3 is significant it implies to an FDR of 0.1% also ruling out that they are randomly co-expressed. (TIF) [file pgen.1011502.s013.tif]

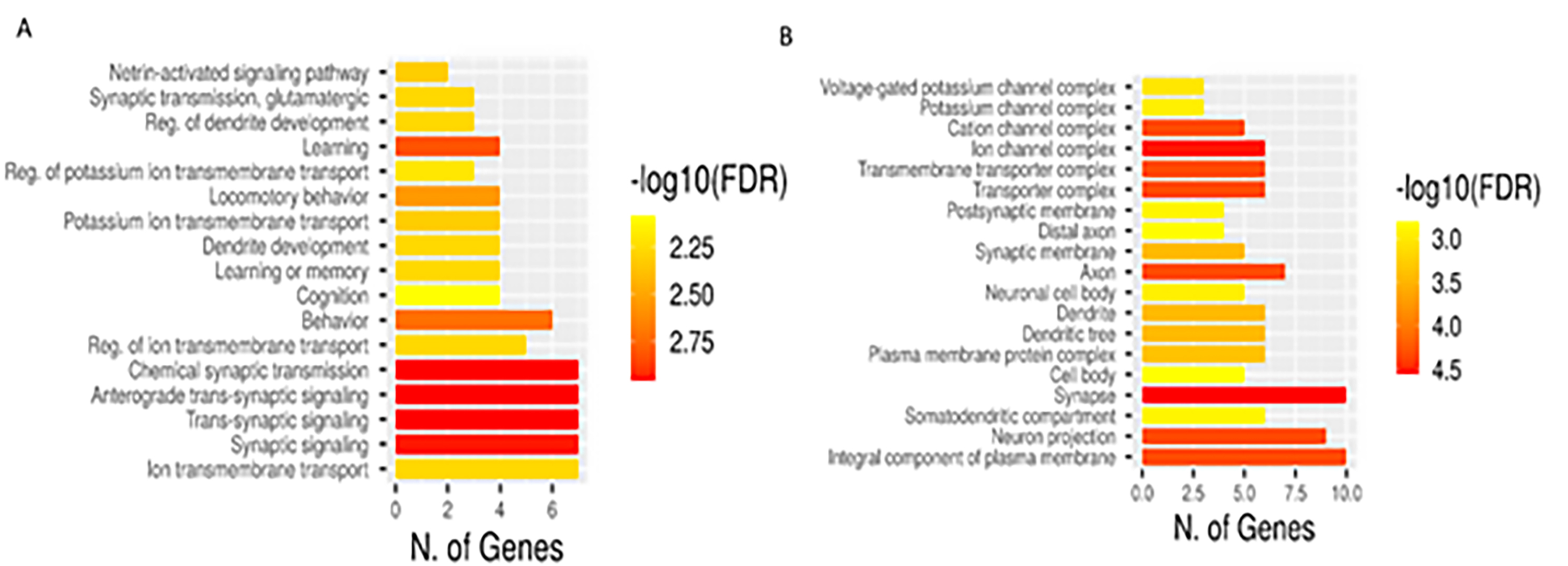

Supplement: S8 Fig — GO Biological Process B. GO Cellular Components. (JPG) [file pgen.1011502.s014.jpg]

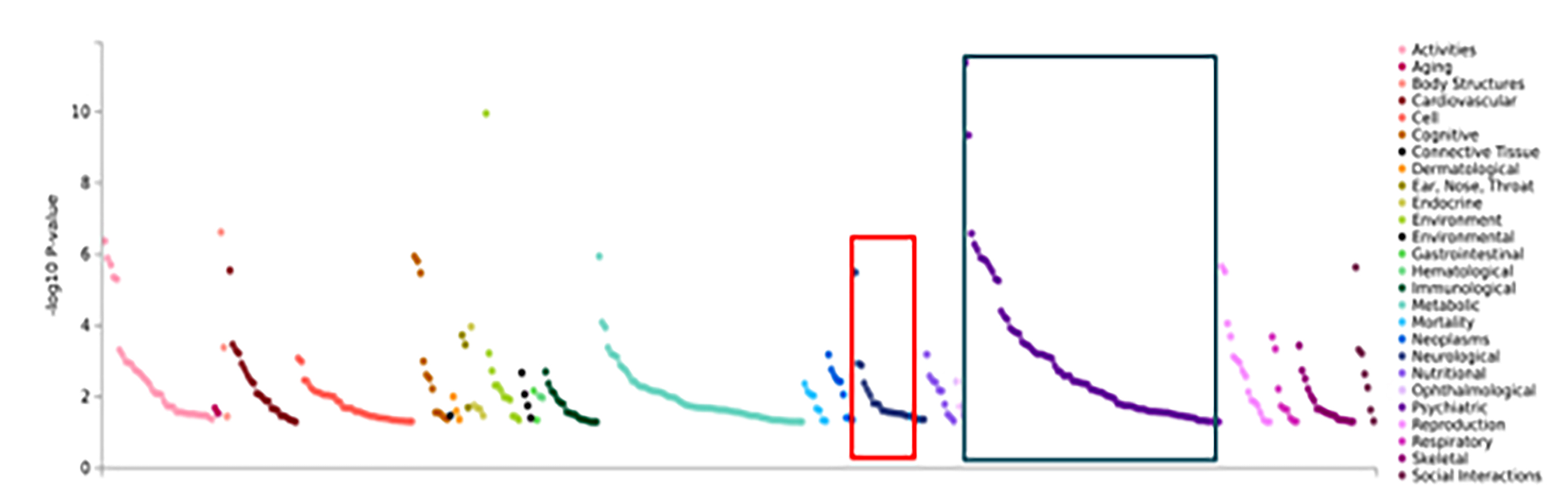

Supplement: S9 Fig — Each point represents a distinct phenotype, with the y-axis showing the -log10(p-value) of the association and different colours indicating various phenotype categories as per the PheWAS catalog. The red box highlights the neurological category, which includes diseases such as Parkinson’s disease and autism. The blue box emphasizes the psychological disease category, featuring conditions like epilepsy and schizophrenia. These specific diseases (Parkinson’s disease, epilepsy, and schizophrenia) are of particular relevance to the findings discussed in the main text of this paper. The plot demonstrates the wide-ranging phenotypic associations of the genetic variant, with a notable concentration of significant associations in neurological and psychological domains. (JPG) [file pgen.1011502.s015.jpg]

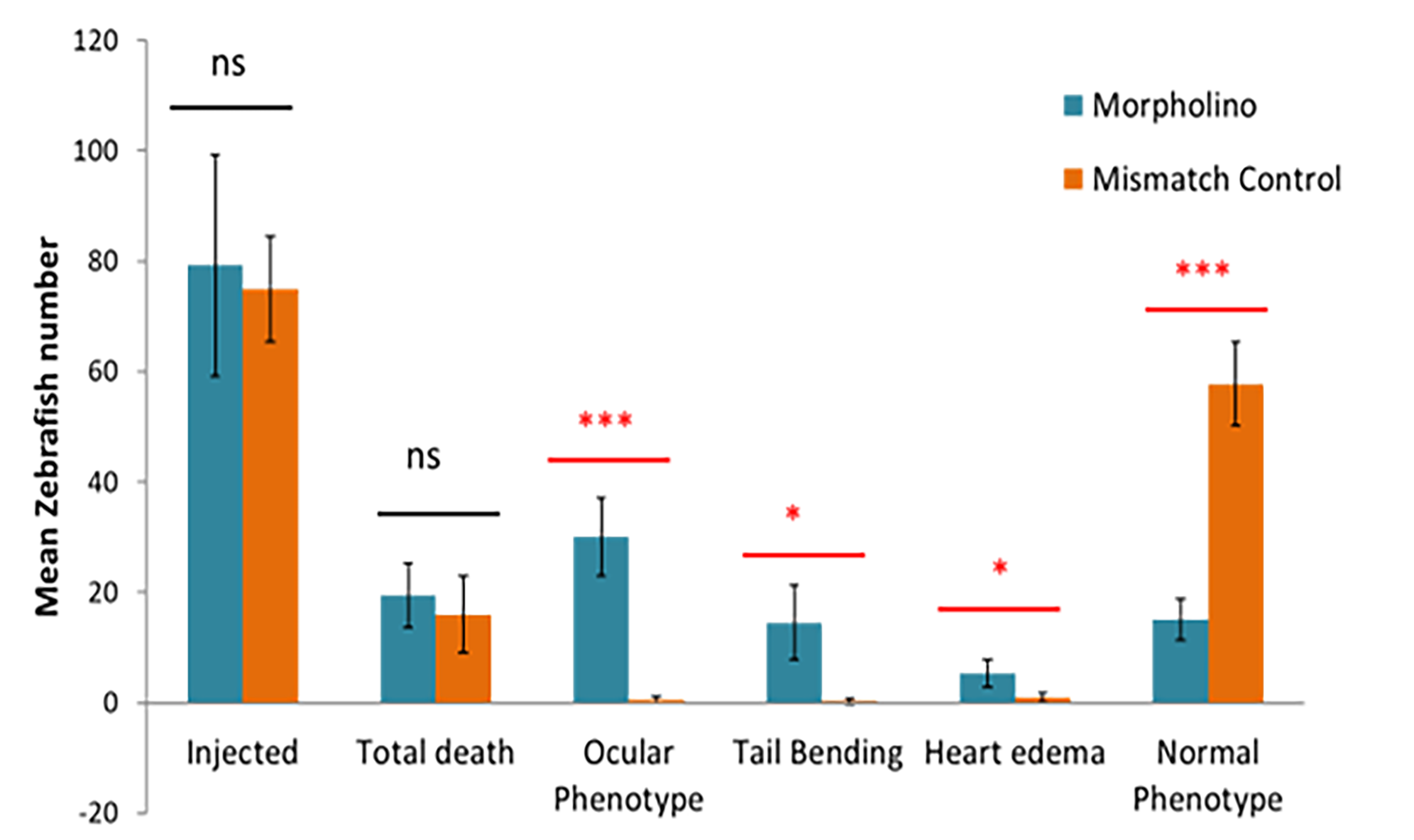

Supplement: S10 Fig — Two tailed student t-test for independent means was used for calculating statistical significance; *p < 0.05, **p < 0.01; ***p < 0.001, ns- = not significant. (TIF) [file pgen.1011502.s016.tif]

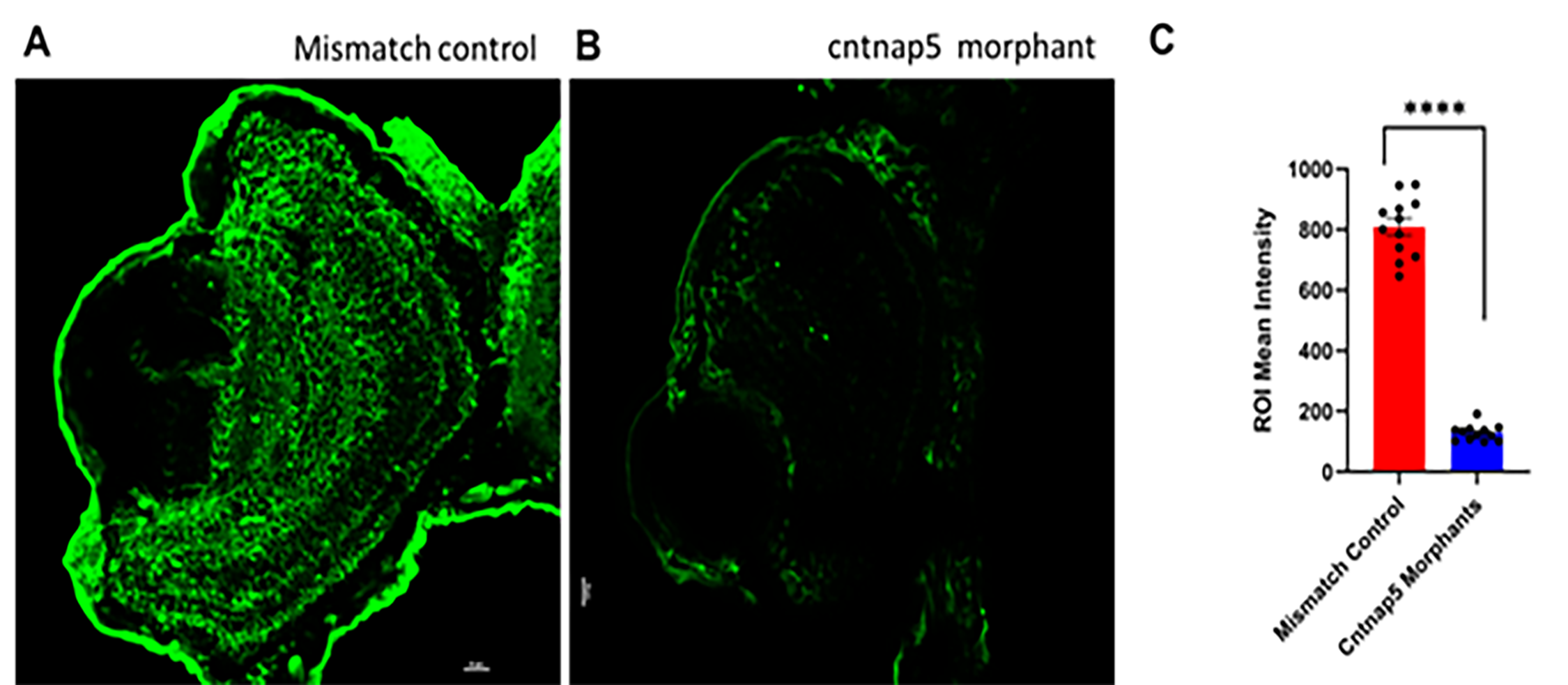

Supplement: S11 Fig — Representative confocal images of cntnap5 expression of eye tissues from mismatch control fish (A) and cntnap5 morphant (B) zebrafish at 96 hpf. C. Comparative analysis of mean intensity of eye for the both groups, bars = mean ± SE, ns not significant, ****p < 0.005. (TIF) [file pgen.1011502.s017.tif]

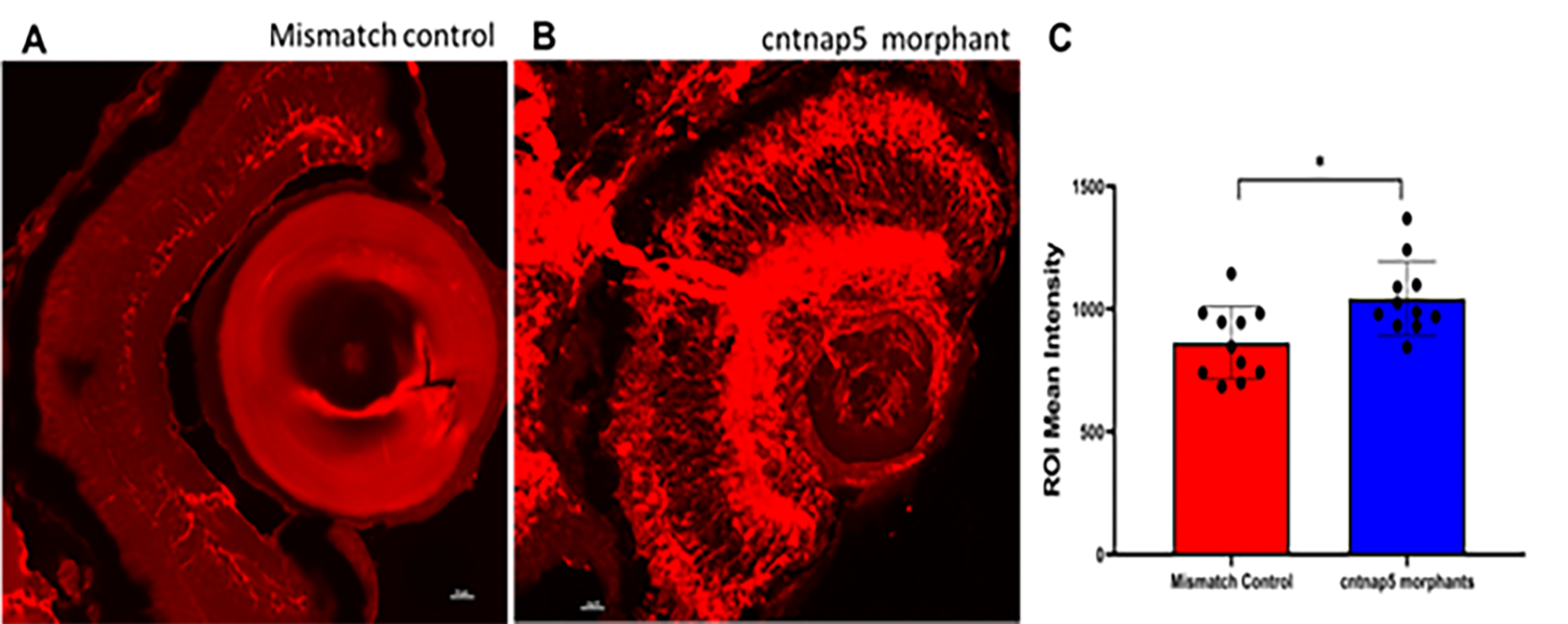

Supplement: S12 Fig — Representative confocal images of acetylated tubulin expression of eye tissues from mismatch control fish (A) and cntnap5 morphant (B) zebrafish at 96 hpf. C. Comparative analysis of mean intensity of eye for the both groups, bars = mean ± SE, ns not significant, ***p < 0.005. (TIF) [file pgen.1011502.s018.tif]

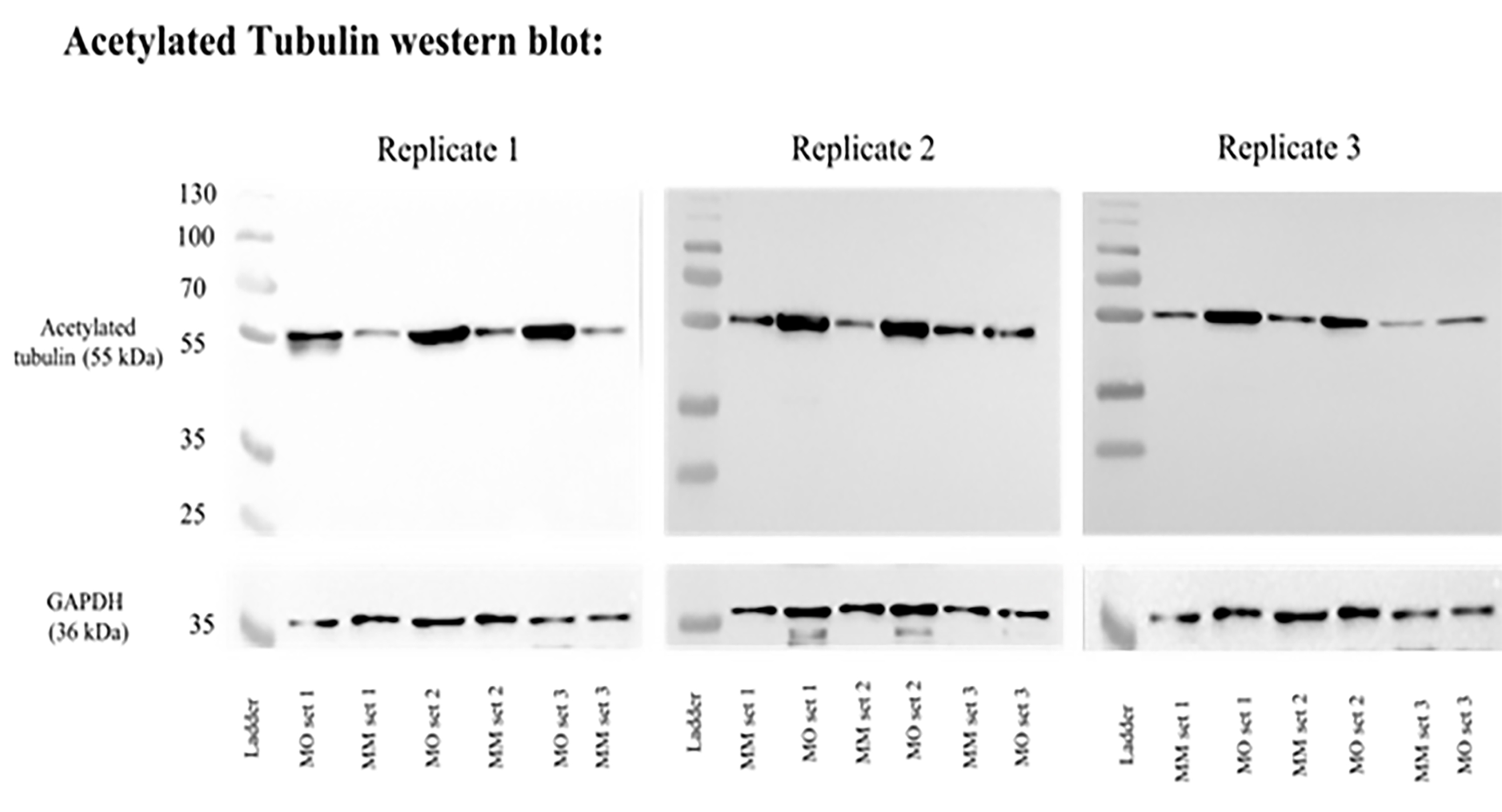

Supplement: S13 Fig — (JPG) [file pgen.1011502.s019.jpg]
