# Supplementary material for: Functional investigation suggests CNTNAP5 involvement in glaucomatous neurodegeneration obtained from a GWAS in primary angle closure glaucoma
Source: PLoS Genet. 2024 Dec 5;20(12):e1011502. doi: 10.1371/journal.pgen.1011502 (PMC11651621; doi:10.1371/journal.pgen.1011502)
Supplement: Attachment — Submitted filename: Response to reviewers comments-final.docx [file pgen.1011502.s021.docx]

**Point-by-point response to Reviewers’ comments**

**Reviewer #1:**

**GWAS, SNP, TSS etc: full forms to be given at first instance of use.**

**Response:** The authors are thankful to the reviewer. In the revised manuscript the full forms of such abbreviations, GWAS (Genome-Wide Association Study), SNP (Single Nucleotide Polymorphism), and TSS (Transcription Start Site), among others, have now been provided when they were first introduced in the manuscript.

**CNTNAP5 has been represented in uppercase and lowercase +/- italics throughout the manuscript. Does it signify a difference in meaning, if not, please maintain consistency.**

**Response:** We regret the inconsistency in the representation of *CNTNAP5* in the manuscript. According to gene nomenclature guidelines, any human gene should be written in uppercase and italicized. For zebrafish ortholog, however, it is written in lower case and italicized. We have followed this throughout the revised manuscript. We ensured that the revised manuscript maintains this convention consistently to avoid confusion.

**Cup disc ratio has been mentioned as a clinical hallmark of glaucomatous optic nerve damage, but neuro-retinal rim damage is the actual hallmark of glaucomatous damage.**

**Response:** We agree with the reviewer that the damage of the neuro-retinal rim is the hallmark of glaucoma. In fact, the damage of the neuro-retinal rim is first detected by measuring the cup-to-disc ratio (CDR). High CDR value (>0.7) essentially indicates thinning of the neuro-retinal rim. In our manuscript, we used cup-to-disc ratio (CDR) as a hallmark, primarily because it is widely used clinically as an indirect indicator of glaucomatous damage, particularly in assessing progression. The CDR is a valuable metric because it represents the relative size of the optic cup compared to the optic disc, which tends to enlarge as glaucomatous damage progresses due to loss of retinal ganglion cells and thinning of the neuro-retinal rim. However, we acknowledge that neuro-retinal rim thinning, observed via imaging techniques like optical coherence tomography (OCT), is indeed a more direct marker of glaucomatous damage. In clinical practice, an increased CDR is often an early sign of structural changes in the optic nerve head and can be detected through fundoscopic examination, making it a convenient and accessible measure for monitoring patients with glaucoma. Studies have shown that changes in CDR correlate with the progression of visual field loss in glaucoma patients, making it a useful marker for assessing disease severity. However, we revised the manuscript to clarify that while CDR is commonly measured, neuro-retinal rim thinning is the actual hallmark of optic nerve damage in glaucoma. This will provide a more nuanced understanding of the pathology. (Please see Ln#83)

**At what stage of the process was ‘Injection of morpholino’ done? The manuscript mentions it after ‘Gross eye measurement’ which doesn’t sound right. For someone from a non genetics background, the timeline of processing of zebrafish embryos would be unclear. Including a flowchart diagram would be a good idea.**

**Response:** The morpholino injection was performed at the single-cell stage, which is a standard procedure to ensure effective gene knockdown throughout developmental stages. "Gross eye measurement" is misnomer, and we apologize for the confusion. In the revised manuscript we clearly stated that morpholino injections were performed at the single-cell stage, followed by morphological eye measurements at 96 hours post fertilization (hpf) (please see Ln#258).

We also appreciate the suggestion to include a flowchart. This will be helpful for readers, especially those from non-genetics backgrounds, to understand the experimental timeline. We have incorporated a flowchart diagram outlining the key steps, including morpholino injection, embryo development, gross morphological measurement, tissue processing, and analysis (please see **- Figure S1**).

**At 96 hours post fertilization, are the zebrafish embryos or larvae? (lines 313 and 335)**

**Response:** Authors are thankful to the reviewer for noticing this. At 96 hours post fertilization (hpf), zebrafish are in the larval stage. By this point, the embryos have hatched, and the larvae are free-swimming. We have corrected the terminology in the manuscript to consistently refer to the zebrafish as larvae at 96 hpf, ensuring accuracy for readers.

**Lines 417 to 420, did all morphant retinas show disorganization, were all 72 eyes thus tested? In comparison, were all mismatch control retinas well organized?**

**Response:** Thank you for this pointer. While we noticed observable defects in all 72 morphant eyes individually (mostly reduction in size, in Figure 3B), we performed detailed analysis of the retina on a representative subset of the histological sections from 6-8 larvae of each batch of injection for each of the two groups (control and morphant), with a total of approx. 35 larvae/group. The analysis showed consistent disorganization in the retinal layers of cntnap5-morphants, compared to the mismatch controls having well-organized retinal layers. Based on these consistent observations across the representative subset, we infer that the majority of the morphant retinas showed similar disorganization (please see Ln#296).

**Comments specific to glaucoma.**

**This study demonstrated reduced vertical and horizontal diameters of eyes of morphant zebrafish larvae. In contrast, in humans, in the presence of congenital glaucoma, the eye is usually enlarged. The vertical and horizontal diameters would be larger in glaucomatous eyes of a child compared to normal eyes.**

**Response:** We appreciate the reviewer bringing up this important distinction. Indeed, in human congenital glaucoma, the condition is typically characterized by buphthalmos, or enlarged eyes, due to elevated intraocular pressure during development. However, our zebrafish model represents a different context. The reduction in vertical and horizontal eye diameters observed in the cntnap5-morphant zebrafish is likely due to disrupted retinal and ocular development resulting from the knockdown of CNTNAP5, which is primarily involved in neurodevelopment and retinal integrity, rather than an elevated intraocular pressure mechanism seen in human congenital glaucoma. The zebrafish model in this study is used to understand the neurodegenerative aspects associated with CNTNAP5 deficiency, not the pressure-related anatomical changes seen in human glaucoma (please see Ln#570).

**Additionally, the morphant eyes showed disorganization of all retinal layers, this is unlike glaucoma where the ganglion cell layer and its axons would be damaged.**

**Response:** Thank you for your insightful comment. We agree that the pattern of damage observed in our zebrafish model differs from the selective loss of retinal ganglion cells (RGCs) and their axons typically seen in human glaucoma. However, the disorganization observed in all retinal layers of the cntnap5-morphant zebrafish is likely due to the developmental role of CNTNAP5 in the organization and integrity of retinal cells. CNTNAP5 is known to play a role in axonal guidance, synaptic stability, and neural development, which may explain the broader structural disruption seen in the morphant retina.

We have identified CNTNAP5 from our previously published GWAS on PACG individuals (Chakraborty S, *et al*, J Biosci. 2021;46:15.). Taking it forward, the aim of the present study is not to directly mimic the specific pathology of human glaucoma, but rather to investigate how CNTNAP5 dysfunction can lead to neurodegenerative changes within the retina, which could provide insights into mechanisms that potentially increase susceptibility to retinal damage in diseases like glaucoma.

We stated this distinction in the manuscript to highlight that the cntnap5-morphant zebrafish model is used to explore the developmental and neurodegenerative role of CNTNAP5 broadly, while recognizing that the specific pattern of damage differs from that seen in human glaucoma (please see Ln#582).

**This study has demonstrated, “The reduction in 520 NeuroTrace intensity specifically in the outer nuclear layer of the retina indicates that photoreceptor neurons are particularly impacted by cntnap5 knockdown.” Photoreceptors are not primarily affected in glaucoma.**

**Response:** Thank you for highlighting this point. We agree that photoreceptors are not the primary site of damage in human glaucoma, where the retinal ganglion cells (RGCs) are typically most affected. However, in our study, the cntnap5-morphant zebrafish model demonstrated a reduction in 520 NeuroTrace intensity across all retinal layers, not just the outer nuclear layer. This suggests that the knockdown of CNTNAP5 impacts the overall structural integrity of the retina, including but not limited to the photoreceptor layer.

We performed a statistical analysis of NeuroTrace intensity for the entire retina, which confirmed a significant decrease in staining across multiple retinal layers in the cntnap5-morphants. This broader reduction in NeuroTrace intensity likely reflects the disrupted retinal architecture due to CNTNAP5 deficiency, indicating that the effects extend beyond the photoreceptor layer alone.

In the revised manuscript, we have acknowledged this finding and clarified that the overall decrease in NeuroTrace intensity suggests a more widespread impact on retinal structure, consistent with the role of CNTNAP5 in maintaining retinal integrity. We appreciate the reviewer's suggestion, which allowed us to refine this interpretation (Ln#590).

**The study has not demonstrated damage at the angle of the anterior chamber angle (as seen in PACG), nor has intraocular pressure been evaluated. At present, what is there to say that the morphant eyes have developed glaucoma and not some other retinal disease?**

**Response:** Thank you for raising this important point. We acknowledge that our study did not focus on evaluating intraocular pressure (IOP) or damage to the anterior chamber angle, both of which are key features of primary angle closure glaucoma (PACG). Our study aimed to investigate the role of CNTNAP5 in retinal neurodegeneration rather than modelling the full spectrum of glaucoma pathology, including angle closure and IOP elevation. The zebrafish model we used specifically focuses on retinal development and degeneration following CNTNAP5 knockdown, rather than replicating the exact mechanisms of PACG. We revised the manuscript to clearly state that our model demonstrates CNTNAP5-related neurodegeneration, which could contribute to retinal disease, but we are not claiming that it fully models the clinical features of PACG, such as anterior chamber angle damage or elevated IOP. This distinction was emphasized to avoid any confusion about the scope of our findings (Ln#640)

**Primary angle closure glaucoma is a disease of middle and older aged individuals. Could this study be more appropriate if evaluation was done on adult zebrafish?**

**Response:** Thank you for this important observation. We fully acknowledge that primary angle-closure glaucoma (PACG) predominantly affects middle-aged and older individuals, where age-related changes in the eye’s anatomy and physiology play a critical role in the disease. Using adult models can indeed offer valuable insights into age-related aspects of glaucoma, such as changes in intraocular pressure (IOP) dynamics and anterior chamber morphology, which are more relevant to the pathophysiology of PACG.

However, our decision to use zebrafish larvae in this study was based on several scientific considerations. First, zebrafish larvae provide a rapid and efficient model to study genetic function, allowing us to observe the developmental role of CNTNAP5 and its impact on retinal structure and integrity at an early stage. The optical transparency of zebrafish larvae is advantageous for *in-vivo* imaging, making it possible to visualize structural changes in the retina and other ocular tissues in detail. This is particularly beneficial when investigating genes like CNTNAP5, which play a role in neural development and organization.

Additionally, zebrafish larvae are highly amenable to gene knockdown using morpholino technology, allowing us to achieve effective CNTNAP5 suppression and assess the resultant phenotypes within a relatively short timeframe. This approach helps to establish a foundational understanding of how CNTNAP5 loss affects retinal organization, which can be crucial before extending the study to adult models.

We agree that future studies using adult zebrafish models would be highly valuable, especially to explore the long-term effects of CNTNAP5 dysfunction on retinal health and to assess features that develop with age, such as changes in IOP or anterior chamber depth that are relevant to PACG. Such studies would complement our findings in larvae, providing a more comprehensive understanding of CNTNAP5’s role across different stages of retinal development and aging.

We have included this point in the discussion section to highlight the rationale for using zebrafish larvae in our study while recognizing the potential benefits of future studies in adult models. This balanced approach will provide readers with a clearer understanding of the strengths and limitations of our model (Ln#643).

**Reviewer #2: Comments by section:**

**Figures:**

**Quality and resolution of figures 1 and 2 is low**

**Response:** Thank you for noticing this. We have enhanced the quality and resolution of Figures 1 and 2 in the revised manuscript, ensuring all details are clearly visible and meet publication standards. The updated high-resolution figures are provided in the revised submission.

**Fig 1D unclear if SNP cluster II is statistically significantly different in p-values than cluster I - add labels to each cluster, add in Bonferroni significance dotted threshold line**

**Response:** Thank you for your valuable feedback. We mentioned, in Figure 1C, two clusters of SNPs are identified based on their genomic locations and association with CREs (cis-regulatory elements). The left cluster (marked by a red box) contains SNPs such as rs780010, rs17011394, rs1430263, rs733112, rs2115890, rs2901264, and rs17011381, which are located in regions with a lower density of CREs. The right cluster includes SNPs such as rs17011429, rs2553628, rs17011420, rs2553625, rs779979, and rs17724018, which are found in regions with a higher abundance of CREs, indicating a higher probability of functional relevance. To maintain parity with the clustering observed in Figure 1C, we have grouped the SNPs into two clusters in Figure 1D as well. This ensures consistency between the figures and provides a clearer visual representation of the SNPs with respect to their p-values and potential regulatory impact. Additionally, to clarify the differences in significance between these clusters, we added a Bonferroni significance threshold line to Figure 1D. This threshold will help readers easily identify which SNPs are statistically significant and how the right cluster—with its high abundance of CREs—compares to the left cluster.

**Fig S3B is a bit confusing, the first half of it does show the long interaction arcs, but then what regions we are looking at in the bottom half of the figure is unclear - distal and proximal interactions are not obvious - provide more context in the caption**

**Response:** Thank you for bringing this to our attention. As suggested by the reviewer, we have now revised the caption to provide a clearer explanation of the figure's components and helps readers understand the context of the proximal and distal interactions shown. It also explains the significance of the different elements in the visualization, making it easier to interpret the long-range chromatin interactions involving the *CNTNAP5* intron (Now in revised version- Figure S4B).

**Missing closing parenthesis in fig S6 caption**

**Response:** We appreciate your careful review. The missing closing parenthesis in the Figure S6 caption has been added in the revised manuscript (Now in revised version- Figure S7).

**Cite phewas catalog when mentioning fig S8 and perhaps modify the figure to show the diseases within the neurological category or label the data points with the relevant diseases mentioned in the text of the paper (epilepsy, schizophrenia, and Parkinson’s disease)**

**Response**: Thank you for your valuable feedback regarding Figure S8. In the revised manuscript, we have cited the PheWAS catalog (Ln#215).

We appreciate the reviewer's suggestion to enhance the clarity of Figure S8. In response, we have modified the figure to highlight the specific diseases mentioned in the text of our paper. Specifically: We have added visual indicators (coloured boxes) to emphasize the neurological and psychological disease categories. The red box now highlights the neurological category, which includes Parkinson's disease, autism and other related conditions. The blue box emphasizes the psychological disease category, which includes epilepsy and schizophrenia. We have updated the figure legend to explicitly mention these disease categories (Now in revised version- Figure S9).

**I would suggest titles mentioned either in the figures for clarity, ex. for fig 4B I would add something like “Acetylated Alpha Tubulin Levels (Whole Embryo)” for clarity**

Thank you for your thoughtful suggestion to improve the clarity of our figures. We have implemented your recommendation as follows:

1. For Figure 4A, we have added the title "Cntnap5 and Acetylated Tubulin Expression in Zebrafish Eye Tissue (96 hpf)" directly on the figure.
2. For Figure 4B, we have added the title "Acetylated Alpha Tubulin Levels (Whole Embryo) (72 hpf)" as you suggested.

We sincerely thank you for this valuable input, which has undoubtedly enhanced the quality and accessibility of our visual data presentation.

**Background + Methods:**

**Not clear how we arrive at the 13 CNTNAP5 intronic and 3’UTR SNPs - these are the output from conditional analysis of the locus and the CRE analysis? Later sentence sounds like the 13 were identified in RegulomeDB. Unclear, please clarify. And if 13 SNPs were identified from the previous GWAS, please provide their information (odds ratio, p-value in a supplemental table)**

**Response:** Thank you for your comment and for the opportunity to clarify this aspect of our study. The 13 SNPs associated with *CNTNAP5* were identified through a conditional analysis of our previous GWAS results, focusing on the *CNTNAP5* locus. These SNPs emerged as significant after conditioning on the lead variants, highlighting them as potentially important genetic contributors to the locus’s overall signal.

To further explore the potential regulatory impact of these SNPs, we performed a RegulomeDB analysis, which provided scores indicating the likelihood that each SNP functions in regulatory elements, such as enhancers or transcription factor binding sites. This allowed us to assess whether the identified SNPs might play a role in gene regulation, which could be relevant to the mechanisms by which these variants contribute to disease risk.

Additionally, as part of the revision, we incorporated eQTL analysis for the 13 SNPs based on data from Dr. Anand Swaroop’s group in NEI, NIH, USA, who kindly shared this data with us. This includes eQTL results using updated QTLtools for integrating eQTLs with mQTLs, as described in their recent publication (https://www.nature.com/articles/s41467-024-46063-8). These eQTL data provide insight into how these SNPs might influence *CNTNAP5* expression levels in the retinal transcriptomic dataset.

We have included a supplemental table in the revised manuscript that provides detailed information on the 13 SNPs, including their odds ratios, p-values from the GWAS (**Table S1**). Thank you again for this important suggestion, which has helped strengthen the manuscript.

**Have you considered overexpression analysis? Has this previously been done for cntnap5 and if so, what are the phenotypes?**

**Response:** Thank you for your suggestion. While our study focused on CNTNAP5 knockdown using morpholino technology to understand the loss-of-function effects in retinal development and neurodegeneration, we agree that overexpression analysis could provide additional insights into the gene’s role. To date, there has been limited investigation into CNTNAP5 overexpression in zebrafish or other models, especially in the context of retinal development or glaucoma. However, previous studies on CNTNAP5 as a neurexin protein in neurological disorders have suggested that abnormal expression levels can affect neuronal communication and axonal function, given its role in synaptic formation and neuronal development Chatterjee M, et al, Neural Regen Res. 2019 Feb;14(2):206-216, Pagnamenta AT et al, Biol Psychiatry. 2010 Aug 15;68(4):320-8

We have added a discussion on the potential value of overexpression studies to further explore CNTNAP5's role, particularly in balancing its effects on retinal and neuronal integrity. Future experiments, including overexpression models, could help clarify whether increased CNTNAP5 expression leads to opposite or compensatory phenotypes compared to the knockdown results. In the revised manuscript, we mentioned that such studies would complement our findings and offer a fuller picture of this gene's role in retinal health and disease (Ln#640).

**Presumably the previous GWAS from which CNTNAP5 was selected was performed in human data - please mention if the CNTNAP5 gene in zebrafish is orthologous with similar function as in humans or if the zebrafish was transgenic with a humanized version of the gene**

**Response:** Thank you for bringing this up. Yes, the CNTNAP5 gene identified in the previous GWAS was based on human data. In zebrafish, cntnap5 is an ortholog of the human CNTNAP5 gene, sharing significant sequence and functional similarity. This zebrafish ortholog of human CNTNAP5 gene allows us to investigate the role of CNTNAP5 in retinal and neuronal development. Our study uses zebrafish with knockdown of the both the orthologs cntnap5 gene- cntnap5a and cntnap5b, not a transgenic line with the humanized version of the gene. We have stated this in the manuscript, explaining the ortholog between the zebrafish and human genes and the rationale for using the zebrafish model to explore the gene’s function (Ln#258).

**Previously cited association of CNTNAP5 with neurological conditions are mentioned but do we know what mechanisms (if any) this gene has been hypothesized to play a role in that leads to dysregulation and disease progression/development?**

**Response:** The association of CNTNAP5 with various neurological conditions, such as autism spectrum disorder, schizophrenia, and epilepsy, has been noted in previous studies. Mechanistically, CNTNAP5 is part of the neurexin family, which is involved in synaptic function, axonal organization, and cell adhesion in the nervous system. Disruption of CNTNAP5 can affect neural connectivity and axonal stability, which may lead to neurodevelopmental defects and neurodegeneration. The precise mechanisms remain under investigation, but it is hypothesized that altered expression of CNTNAP5 disrupts synaptic architecture and neural circuit integrity, contributing to disease progression. We expanded this section of the manuscript to include a discussion of these mechanisms, providing context for its role in neurological diseases and its potential contribution to retinal neurodegeneration observed in our study (Ln#609).

**Additional details behind the intuition of mismatched controls would be helpful as general knowledge for the reader.**

**Response:** Thank you for the suggestion. The mismatched controls in our study refer to morpholino sequences that differ by five base pairs from the cntnap5-targeting morpholinos. These controls are essential for ruling out off-target or non-specific effects of morpholino injection. By using a mismatch control, we ensure that any phenotypic differences observed (such as retinal disorganization and neurodegeneration) are specifically due to the knockdown of cntnap5 and not to non-specific effects of morpholino treatment. We have added more details in the methods section, explaining the rationale behind the mismatched control and its importance in validating the specificity of the gene knockdown experiments for readers unfamiliar with this technique (Ln#265).

**Discussion**

**16. I find the discussion section difficult to read and parse as it is discussing many different results one after another. I would like to see a restructuring so that the main overarching points are discussed first, with the results mentioned as supporting details that can be further delved into. For example, having a zoomed-out view in the first part of the discussion will be helpful to orient the reader to the main aims and findings of the study. This can then be broken down into 1) the broader implications of revealing glaucoma genetics, 2) the molecular genetic underpinnings of the disease, and 3) finally the in vitro analysis to support the hypothesized mechanisms of action. (i.e. Line 501 onwards reads as though it could be the beginning of the discussion, giving us the larger picture and then breaking it down to specific biology.)**

**Response:** Thank you for this valuable feedback. We appreciate your suggestion for a more structured and reader-friendly approach. Accordingly, we have revised the discussion beginning with a zoomed-out view that clearly presents the main aims and overarching findings of the study, helping to orient the reader. This introductory section summarized the key implications of our research, such as identifying CNTNAP5 as a novel genetic contributor to glaucoma-related neurodegeneration and demonstrating its role in retinal development.

After this broader introduction, we restructured the discussion into three thematic sections:

**Revealing the broader implications of glaucoma genetics:** Here, we focused on how our findings contribute to understanding the genetic risk factors associated with glaucoma, particularly PACG, and how these insights could inform future research and therapeutic strategies.

**Molecular genetic underpinnings of the disease:** In this section, we explored the specific molecular mechanisms by which CNTNAP5 and related pathways might contribute to neurodegenerative processes in glaucoma. We also discussed how these findings align with or extend previous research on the gene's role in neurological conditions.

**In vivo analysis to support the hypothesized mechanisms of action:** Finally, we highlighted how our in vivo and zebrafish model analyses provide functional evidence supporting the role of CNTNAP5 in retinal neurodegeneration. This included detailed discussions on the results from the morpholino knockdown, retinal disorganization, and behavioral assays, as well as their relevance to the disease model.

We followed the suggestion to use Line 513 onwards as the starting point of the discussion, as it provides a good summary of the larger picture, and we then moved into more detailed results to support the broader narrative. This restructuring made the discussion more coherent and reader-friendly while clearly linking each result to the study's overall objectives and contributions to the field.

Thank you again for this helpful suggestion; we believe these changes significantly improved the clarity and impact of the discussion section.

**I would also like to see more discussion of the results beyond stating the molecular interpretation of the individual result.**

**Response:** Thank you for your suggestion. We agree that the discussion would benefit from a more in-depth exploration of the broader implications of the results, beyond just the molecular interpretations. In the revised discussion, we expanded on how our findings contribute to the overall understanding of glaucoma and neurodegenerative diseases. Specifically, we emphasized the potential translational impact of identifying CNTNAP5 as a risk gene, the relevance of our zebrafish model to human retinal neurodegeneration, and how our results may inform future genetic or therapeutic research for glaucoma and other neurodegenerative conditions. This expanded discussion helped place the molecular findings in a wider context, giving readers a better sense of the study's impact.

**You may consider adding headings into the discussion as well that can help organize the conversation based on different axes of analysis, and then also separate out the limitations and conclusions**

**Response:** We appreciate your suggestion to introduce headings in the discussion section. We have added headings that clearly separate different axes of analysis, such as:

- **Broader Implications for Glaucoma Genetics**
- **Molecular genetic underpinnings of the disease**
- **In vivo analysis to support the hypothesized mechanisms of action**
- **Conclusions (limitations included)**

These headings made it easier for readers to follow the discussion and understand the flow of the narrative. Thank you so much.

**I would recommend including an overarching figure of a hypothesized mechanism of action; this would be very helpful for the reader to put together the rest of the analyses that were done to unveil different aspects of function. Please note that the graphical abstract does provide some level of overview, but additional text could be provided between the arrows to more clearly indicate the steps of the study.**

**Response:** We agree that an overarching figure illustrating the hypothesized mechanism of action would be valuable for readers. We created a detailed figure that outlined the functional role of CNTNAP5 in retinal development and neurodegeneration, linking the various stages of our study. This figure included additional explanatory text between the arrows to clearly show the sequential steps of our experimental workflow, including the molecular mechanisms we hypothesized are involved in CNTNAP5-related retinal neurodegeneration. This provided a visual summary that tied together the different analyses presented in the manuscript. We ensured that this figure complemented the graphical abstract while offering more specific details about the mechanisms and findings of the study. (Please see the graphical abstract)

**Missing period on line 546**

**Response:** Thank you for pointing this out. We have corrected the typographical error by adding the missing period on line 546 (Now in Ln#617). We also carefully proofread the entire revised manuscript to ensure that no such error exists.

**Conclusion**

**Given that the target was identified in a GWAS, can you elaborate on how your results should be interpreted in the context of human biology? Are the pathways and mechanisms discussed consistent in humans as well or are these results to be viewed specifically with respect to zebrafish?**

**Response:** Thank you for this thoughtful comment. The CNTNAP5 gene was identified through a GWAS in human, and our zebrafish model was used to explore its role in retinal development and neurodegeneration. While zebrafish provide an effective system for studying the basic functions of CNTNAP5, we agree that it is crucial to interpret the findings within the context of human biology.

Many of the pathways and mechanisms affected by CNTNAP5 in zebrafish, such as neural development, synaptic architecture, and axonal organization, are conserved between zebrafish and humans. The neurodegenerative phenotypes observed in our study align with the gene's proposed role in human neurological diseases, such as autism spectrum disorder, schizophrenia, and epilepsy. However, some aspects of the zebrafish model, such as retinal development and the specific cellular architecture of the eye, may differ from human biology.

We revised the discussion to address the conservation of CNTNAP5's function in humans, focusing on shared pathways, such as neural circuit organization and cytoskeletal stability, which are relevant in both species. Additionally, we emphasized that while zebrafish provide a valuable model to investigate the gene's fundamental biological roles, further validation in human cells or tissues is necessary to fully understand how the mechanisms we observed contribute to disease processes like glaucoma in humans (Ln#640).

This will help position our findings within a broader context, highlighting both the translational relevance and the need for complementary studies in human systems.
